# Short-term exposure to fine particulate air pollution and emergency department visits for kidney diseases in the Atlanta metropolitan area

**DOI:** 10.1097/EE9.0000000000000164

**Published:** 2021-08-06

**Authors:** Jianzhao Bi, Vaughn Barry, Ethel J. Weil, Howard H. Chang, Stefanie Ebelt

**Affiliations:** a Department of Environmental & Occupational Health Sciences, School of Public Health, University of Washington, Seattle, Washington; b Gangarosa Department of Environmental Health, Rollins School of Public Health, Emory University, Atlanta, Georgia; c Department of Medicine, School of Medicine, Emory University, Atlanta, Georgia; d Department of Biostatistics and Bioinformatics, Rollins School of Public Health, Emory University, Atlanta, Georgia

**Keywords:** PM_2.5_ total mass, PM_2.5_ components, Criteria gases, Time-series, Renal diseases, Single-day lags, Distributed lags

## Abstract

Toxicological evidence has shown that fine particulate matter (PM_2.5_) may affect distant organs, including kidneys, over the short term. However, epidemiological evidence is limited.

**Objectives:**

We investigated associations between short-term exposure to PM_2.5_, major PM_2.5_ components [elemental carbon (EC), organic carbon (OC), sulfate, and nitrate], and gaseous co-pollutants (O_3_, CO, SO_2_, NO_2_, and NO_x_) and emergency department (ED) visits for kidney diseases during 2002–2008 in Atlanta, Georgia.

**Methods:**

Log-linear time-series models were fitted to estimate the acute effects of air pollution, with single-day and unconstrained distributed lags, on rates of ED visits for kidney diseases [all renal diseases and acute renal failure (ARF)], controlling for meteorology (maximum air and dew-point temperatures) and time (season, day of week, holidays, and long-term time trend).

**Results:**

For all renal diseases, we observed positive associations for most air pollutants, particularly 8-day cumulative exposure to OC [rate ratio (RR) = 1.018, (95% confidence interval [CI]: 1.003, 1.034)] and EC [1.016 (1.000, 1.031)] per interquartile range increase exposure. For ARF, we observed positive associations particularly for 8-day exposure to OC [1.034 (1.005, 1.064)], EC [1.032 (1.002, 1.063)], nitrate [1.032 (0.996, 1.069)], and PM_2.5_ [1.026 (0.997, 1.057)] per interquartile range increase exposure. We also observed positive associations for most criteria gases. The RR estimates were generally higher for ARF than all renal diseases.

**Conclusions:**

We observed positive associations between short-term exposure to fine particulate air pollution and kidney disease outcomes. This study adds to the growing epidemiological evidence that fine particles may impact distant organs (e.g., kidneys) over the short term.

## Abstract 2

Supplemental Digital Content is available in the text.

**What this study adds**

Most of the PM_2.5_-related short-term epidemiological studies have focused on cardiorespiratory diseases. Although emerging toxicological evidence shows PM_2.5_ may affect distant organs, including kidneys, over the short term, epidemiological evidence is limited. To our knowledge, this is the first time-series study to explore associations between short-term exposure to PM_2.5_, major PM_2.5_ components, and gaseous co-pollutants and kidney disease outcomes in the United States. This study observed positive associations between short-term exposure to PM_2.5_ and kidney disease outcomes, which expanded the current understanding of the effects of short-term PM_2.5_ exposure on body systems other than the cardiorespiratory system.

## Introduction

Fine particulate matter with an aerodynamic diameter less than or equal to 2.5 micrometers (PM_2.5_) has caused severe pollution issues worldwide and contributed to a substantial global health burden.^1,2^ A majority of PM_2.5_-related health studies have focused on its impacts on mortality and respiratory disease, stroke, and cardiovascular disease morbidity.^3,4^ However, emerging evidence has shown that inhaled fine particles may affect distant organs by passing across the alveoli and/or releasing inflammatory mediators into circulation.^5–8^ Specifically, inhaled nanoparticles have been detected in urine within minutes after exposure,^9^ demonstrating that these particles can pass through kidneys and may thus directly affect kidney function.

A growing body of epidemiological research has reported associations between long-term exposure to PM_2.5_ and a variety of renal disease outcomes.^10–14^ For example, Mehta et al^13^ observed that long-term exposure to PM_2.5_ led to kidney function decline with a lower estimated glomerular filtration rate (eGFR). Bowe et al^14^ demonstrated positive associations between long-term exposure to PM_2.5_ and increased risks of incident chronic kidney disease (CKD), eGFR decline, and end-stage renal disease (ESRD). Plausible mechanisms of these associations have also been explored. Toxicological evidence revealed that pulmonary exposure to PM_2.5_, such as those in diesel exhaust, could potentiate adenine-induced^15^ and cisplatin-induced renal diseases.^16–19^ For example, Waly et al^17^ found that exposure to diesel exhaust particles (DEPs) exacerbated the effects of cisplatin-induced toxicity in human embryonic kidney cells 293 (HEK-293). Nemmar et al^15^ discovered that pulmonary exposure to DEPs aggravated adenine-induced chronic renal failure in mice with increased renal oxidative stress, inflammation, and DNA damage. Although the mechanistic plausibility of PM_2.5_ reaching the kidney within a relatively short time is clear,^9^ limited epidemiological studies have been conducted to examine whether and to what extent short-term exposure to PM_2.5_ is associated with kidney disease outcomes.^20–23^

In this analysis, we investigated associations between short-term exposure to PM_2.5_, major PM_2.5_ components, and gaseous co-pollutants and emergency department (ED) visits for kidney diseases in Atlanta, Georgia during 2002–2008 using a population-based time-series approach. The study built off a large body of existing work on the impacts of air pollution on ED visit outcomes in Atlanta that has focused predominantly on respiratory and cardiovascular disease impacts.^24–31^

## Methods

### Emergency department visits data

ED visit billing records were collected for metropolitan Atlanta health facilities (N of facilities = 48) for the period of 2002–2008 from the Georgia Hospital Association. The ED visit records included information regarding the patient’s age, gender, and race, date of ED visit, Zone Improvement Plan (ZIP) code of patient residence, and International Classification of Diseases, ninth Revision (ICD-9) diagnosis codes. ED visits for all renal diseases and acute renal failure (ARF) were identified *a priori* using ICD-9 codes 580–593 and 584, respectively; visits were identified via primary ICD-9 code only as well as any (i.e., primary, secondary, tertiary, and up to 50^th^) ICD-9 code. ESRD patients on dialysis (ICD-9: 585.6) and chronic kidney disease patients with unspecified disease status (ICD-9: 585.9) were not included because short-term exposure to air pollution is unlikely to further worsen the kidney function of patients already on dialysis (with less than 5% function remaining). Multiple visits by the same patient on the same day were only counted once (N of ED visits = 306,595). Patient-level ED visits for all renal diseases and ARF were aggregated to daily counts (N of days = 2,557) for outcomes defined based on primary as well as any ICD-9 codes. The Emory University Institutional Review Board approved this study and granted an exemption from informed consent requirements, given the minimal risk nature of the study and the infeasibility of obtaining informed consent from individual patients.

### Ambient air pollution and meteorological data

Ambient air pollution exposure estimates were based on fusing ground-level air pollution measurements and simulations from the Community Multi-Scale Air Quality (CMAQ) model, as described previously.^32,33^ The fusion of simulated data and ground-level measurements may help reduce exposure measurement error that can arise from spatiotemporal variation in concentrations not captured by measurements from air quality stations.^34,35^ The fused air pollution data had a 12-km spatial resolution with spatiotemporal complete coverage. We then estimated the pollution concentrations for each ZIP Code Tabulation Area (ZCTA) using the 2010 Census Bureau boundaries by determining the fraction of a ZCTAs area within each 12-km grid cell and area weighting the observation-simulation data fusion estimates to obtain the ZCTA-specific value.^33^ Finally, we generated time-series of region-wide pollution concentrations by calculating the population-weighted averages of individual ZCTA-specific values. The air pollutants included PM_2.5_, major PM_2.5_ components [elemental carbon (EC), organic carbon (OC), sulfate, and nitrate], and gaseous co-pollutants (O_3_, CO, SO_2_, NO_2_, and NO_x_). The selected gaseous co-pollutants are important PM_2.5_ precursors.^36^ The daily metrics of the air pollutants were 8-hour maximum O_3_, 1-hour maximum CO, NO_2_, NO_x_, and SO_2_, and 24-hour average PM_2.5_ and major components (Table 1). The region-wide time-series of daily pollution concentrations were shown in Figure S1; http://links.lww.com/EE/A147.

**Table 1.** Summary statistics for air pollution concentrations, meteorological parameter values, and ED visit counts for kidney diseases during the study period of 2002–2008 (2,557 days)

	Parameter	Mean	Standard deviation	Minimum	Maximum	IQR
Fine particle components	24-hour Avg PM_2.5_, μg/m^3^	15.41	7.12	2.46	61.28	8.99
	24-hour Avg EC, μg/m^3^	1.10	0.59	0.14	4.53	0.68
	24-hour Avg OC, μg/m^3^	2.99	1.54	0.58	21.91	1.75
	24-hour Avg Sulfate, μg/m^3^	4.50	2.99	0.44	21.25	3.52
	24-hour Avg Nitrate, μg/m^3^	0.63	0.57	0.02	5.45	0.60
Criteria gases	8-hour Max O_3_, ppb	42.15	17.34	5.84	109.08	27.04
	1-hour Max CO, ppm	0.65	0.29	0.18	2.07	0.33
	1-hour Max SO_2_, ppb	10.05	6.90	0.53	44.81	8.76
	1-hour Max NO_2_, ppb	21.58	6.97	3.18	45.29	9.52
	1-hour Max NO_x_, ppb	45.63	30.48	4.75	219.46	33.51
Meteorology	Dew-point temperature, °F	49.85	16.49	2.30	74.50	27.40
	Maximum temperature, °F	72.20	14.60	30.00	104.00	24.00
	Minimum temperature, °F	53.54	15.08	8.00	82.00	27.00
Kidney disease ED visits	All renal diseases, primary diagnosis, daily visit counts	52.7	12.3	22.0	94.0	17.0
	All renal diseases, any diagnosis, daily visit counts	119.9	30.5	52.0	216.0	47.0
	Acute renal failure, primary diagnosis, daily visit counts	6.3	3.8	0	23.0	6.0
	Acute renal failure, any diagnosis, daily visit counts	30.2	15.6	2.0	86.0	24.0

Meteorological data on daily maximum air temperature and mean dew-point temperature were obtained from the weather station at the Hartsfield–Jackson Atlanta International Airport, which was close to the urban center of the Atlanta metropolitan area. Thomas et al^37^ compared the temperature measurements at the airport to the region-wide means of a well-known meteorology product (Daymet) in Atlanta and observed a high correlation between the two. For this analysis, we opted to use the airport measurements due to their high-quality, complete temporal coverage, and its central location in our study area with little variation in elevation.

### Statistical analysis

Quasi-Poisson log-linear time-series models were used to investigate the relative risk of ED visits for all renal diseases and ARF associated with short-term exposure to PM_2.5_ and other air pollutants. We examined the effect of air pollution exposure over 8 days, which was motivated by previous findings suggesting that the effect of PM_2.5_ exposure may last over a week-long period.^30,38,39^ For the association between each health outcome and air pollutant, we fitted (1) single-day lag models, where exposures at lag 0 (the same day), lag 1 (the previous day), and up to lag 7 were assessed one at a time, to examine patterns of the relative risk at different lag days and (2) unconstrained distributed lag models to determine the cumulative effect of air pollution exposure over lags 0 to 7.^40^ For the distributed lag model, even though correlation between air pollution concentrations on days close together will exhibit collinearity, the summation of the individual coefficients will be an unbiased estimate of the cumulative effect of a unit increase in pollution over multiple days.^41^ Models were also fitted separately for outcomes defined using primary ICD-9 codes or any ICD-9 codes (primary, secondary, and up to 50^th^). Although the primary diagnosis can reflect more urgent cases of kidney diseases that reflect the primary reason for the visit, any diagnosis can capture a broader range of kidney-related outcomes, including potentially important comorbid conditions. The quasi-Poisson log-linear time-series models were fitted based on the R (ver. 3.5.2) package “dlnm” (ver. 2.3.9).^42,43^

The time-series models were adjusted for the following variables: cubic terms for daily maximum air temperature and mean dew-point temperature, indicator variables for season (spring, summer, fall, and winter), day of week and holidays, and hospital participation (accounted for the entry and exit of hospitals during the study period, which impact ED visit counts over time). The moving averages of daily maximum air temperature and mean dew-point temperature from lag 0 to lag 7 were used in the distributed lag models, and the single-day values of these parameters were used in the single-day lag models. Cubic splines for calendar dates with monthly knots were included to account for long-term time trends and seasonality. Some interaction terms were also added, including the interactions between day of week and season and between daily maximum air temperature (with cubic terms) and season.^30^ The rate ratios (RRs) with 95% confidence intervals (CIs) based on per interquartile range (IQR) increase in the levels of air pollutants were reported.

We conducted sensitivity analyses (SAs) focusing on temperature adjustment since high-temperature has been found to be associated with increased ED visits for kidney diseases in Atlanta.^44^ Five different specifications for temperature adjustment were assessed, as described in Table S1; http://links.lww.com/EE/A147. Results of these sensitivity analyses were compared to those from the main models. Additionally, we also conducted SAs based on two-pollutant models, where PM_2.5_ was treated as the main pollutant and criteria gases (O_3_, CO, SO_2_, NO_2_, and NO_x_) were treated as co-pollutants in each two-pollutant model one at a time. The two-pollutant models were distributed lag models for lag 0-7 with ED visits of any diagnosis for all renal diseases and ARF.

## Results

### Descriptive analyses

Table 1 summarizes ED visits for kidney diseases, air pollution concentrations, and meteorological parameters during the study period. PM_2.5_ had a mean concentration of 15.41 μg/m^3^ during the study period. This level exceeds the primary National Ambient Air Quality Standards (NAAQS) of 12 μg/m^3^ for the annual mean concentration. Major PM_2.5_ components—EC, OC, sulfate, and nitrate—had mean concentrations of 1.10, 2.99, 4.50, and 0.63 μg/m^3^ during the study period, respectively. The criteria gases—O_3_, CO, SO_2_, NO_2_, and NO_x_—had mean concentrations of 42.15 ppb, 0.65 ppm, 10.05 ppb, 21.58 ppb, and 45.63 ppb during the study period, respectively.

### Relative risk estimates of emergency department visits for all renal diseases

**Figure 1.**
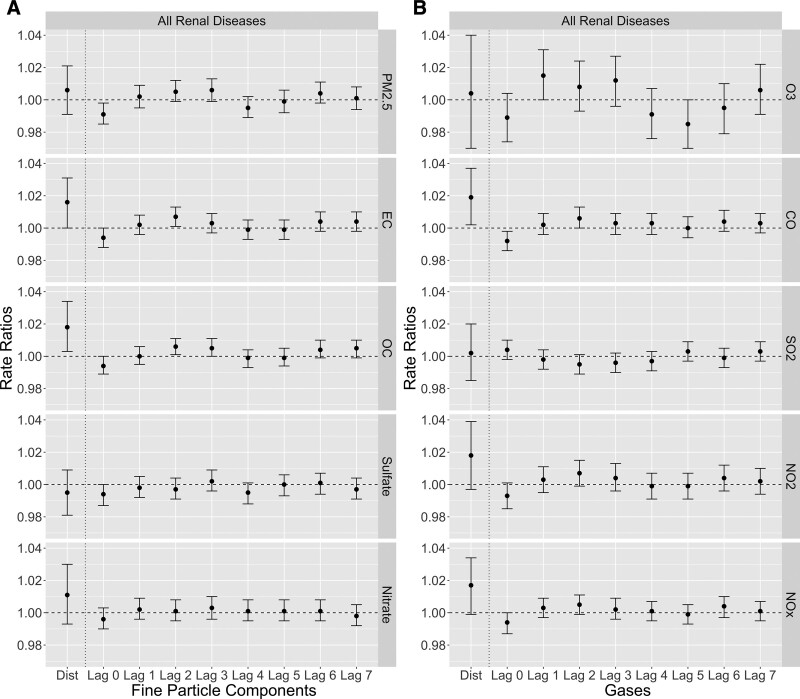
RR estimates of ED visits (any diagnosis) for all renal diseases associated with short-term air pollution exposure (A, fine particle components; B, criteria gases) with different lag structures (distributed lag [dist.] and single-day lags) in Atlanta during the period of 2002–2008.

Figure 1 shows the RR estimates of ED visits (any diagnosis) for all renal diseases associated with short-term exposure to air pollutants with different lag structures. Figure S2; http://links.lww.com/EE/A147 shows the RR estimates of primary ED visits for all renal diseases. Table 2 summarizes the RRs with 95% CIs of the distributed lag models, and Table S2; http://links.lww.com/EE/A147 summarizes the RRs with 95% CIs of the single-day lag models. Generally, the distributed lag models showed stronger health associations than the single-day lag models, suggesting that, as expected, multiple-day exposure to air pollution was associated with a higher risk of all renal diseases than single-day exposure. For single-day lags, lags 2 and 3 tended to have higher RRs than other lags for most of the pollutants except for SO_2_ and its secondary pollutant, sulfate.

**Table 2.** Distributed lag RRs and 95% CIs of the associations between short-term exposure to air pollution and ED visits for kidney diseases

Pollutant	IQR	All renal diseases, any diagnosis RR (95% CI)	All renal diseases, primary diagnosis RR (95% CI)	ARF, any diagnosis RR (95% CI)	ARF, primary diagnosis RR (95% CI)
Fine particle components					
PM_2.5_	8.99 μg/m^3^	1.006 (0.991, 1.021)	1.006 (0.985, 1.028)	1.026 (0.997, 1.057)^a^	1.011 (0.950, 1.076)
EC	0.68 μg/m^3^	1.016 (1.000, 1.031)^a^	1.016 (0.993, 1.040)	1.032 (1.002, 1.063)^b^	1.024 (0.959, 1.093)
OC	1.75 μg/m^3^	1.018 (1.003, 1.034)^b^	1.009 (0.987, 1.032)	1.034 (1.005, 1.064)^b^	0.995 (0.935, 1.059)
Sulfate	3.52 μg/m^3^	0.995 (0.981, 1.009)	1.000 (0.981, 1.021)	1.019 (0.991, 1.047)	1.037 (0.978, 1.100)
Nitrate	0.60 μg/m^3^	1.011 (0.993, 1.030)	1.016 (0.989, 1.045)	1.032 (0.996, 1.069)^a^	1.032 (0.954, 1.117)
Criteria gases					
O_3_	27.04 ppb	1.004 (0.970, 1.040)	0.986 (0.936, 1.037)	1.078 (1.005, 1.158)^b^	1.054 (0.905, 1.227)
CO	0.33 ppm	1.019 (1.002, 1.037)^b^	1.013 (0.987, 1.039)	1.028 (0.994, 1.063)	1.025 (0.951, 1.105)
SO_2_	8.76 ppb	1.002 (0.985, 1.020)	1.018 (0.992, 1.044)	1.013 (0.979, 1.047)	1.024 (0.952, 1.101)
NO_2_	9.52 ppb	1.018 (0.997, 1.039)	1.022 (0.991, 1.054)	1.018 (0.979, 1.059)	1.013 (0.929, 1.105)
NO_x_	33.51 ppb	1.017 (0.999, 1.034)^a^	1.021 (0.995, 1.048)	1.027 (0.993, 1.062)	1.019 (0.946, 1.099)

^a^0.05 ≤ *P* < 0.10.

^b^*P* < 0.05.

For all renal diseases, we observed positive associations (RR > 1.0) for all pollutants except for sulfate. The strongest association was for 8-day CO exposure [RR = 1.019 (95% CI: 1.002, 1.037) per 0.33 ppm increase]. Other strong positive associations included 8-day exposure to OC [1.018 (1.003, 1.034) per 1.75 μg/m^3^ increase], NO_2_ [1.018 (0.997, 1.039) per 9.52 ppb increase], NO_x_ [1.017 (0.999, 1.034) per 33.51 ppb increase], EC [1.016 (1.000, 1.031) per 0.68 μg/m^3^ increase], and nitrate [1.011 (0.993, 1.030) per 0.60 μg/m^3^ increase]. The observed negative association (RR < 1.0) for the 8-day exposure to sulfate (not statistically significant at an alpha level of 0.05) might occur by chance due to large uncertainty. The primary diagnosis of all renal diseases also had positive associations with 8-day exposure to most of the air pollutants (Figure S2; http://links.lww.com/EE/A147). The scales of these associations were comparable to that of any diagnosis of all renal diseases.

### Relative risk estimates of emergency department visits for acute renal failure

**Figure 2.**
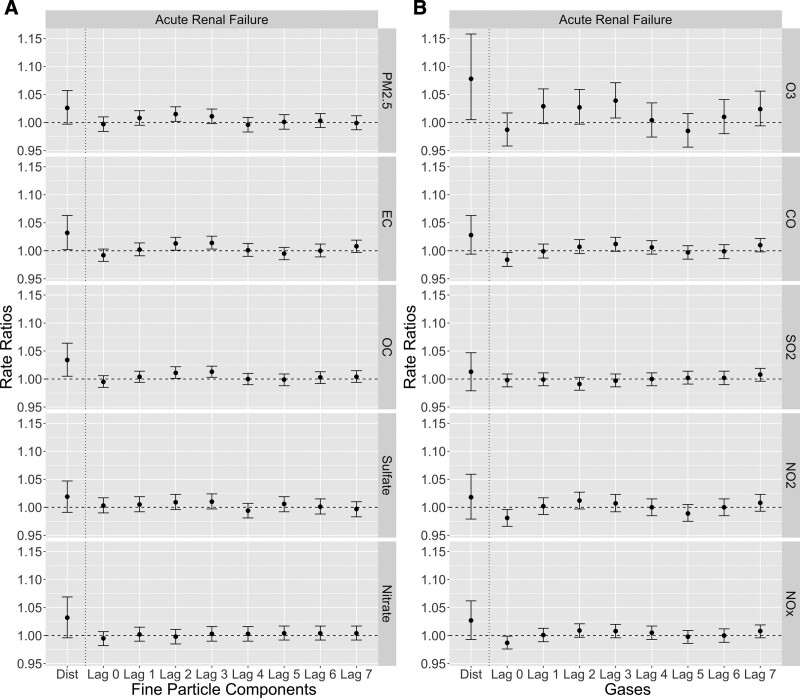
RR estimates of ED visits (any diagnosis) for ARF associated with short-term air pollution exposure (A, fine particle components; B, criteria gases) with different lag structures (distributed lag [dist.] and single-day lags) in Atlanta during the period of 2002–2008.

Figure 2 shows the RR estimates of ED visits (any diagnosis) for ARF associated with short-term exposure to air pollutants with different lag structures. Figure S3; http://links.lww.com/EE/A147 shows the RR estimates of primary ED visits for ARF. Table 2 summarizes the RRs with 95% CIs of the distributed lag models, and Table S2; http://links.lww.com/EE/A147 summarizes the RRs with 95% CIs of the single-day lag models. As is the pattern shown in Figure 2, the distributed lag models showed stronger associations than the single-day lag models. Unlike all renal diseases, lag 7 tended to have higher estimates for the associations for single-day lags of ARF in addition to lags 2 and 3.

The estimated RRs of ED visits for ARF were in general higher than those of ED visits for all renal diseases. The strongest positive association was for 8-day O_3_ exposure [RR = 1.078 (95% CI: 1.005, 1.158) per 27.04 ppb increase]. Other strong positive associations included 8-day exposure to OC [1.034 (1.005, 1.064) per 1.75 μg/m^3^ increase], EC [1.032 (1.002, 1.063) per 0.68 μg/m^3^ increase], nitrate [1.032 (0.996, 1.069) per 0.60 μg/m^3^ increase], and PM_2.5_ total mass [1.026 (0.997, 1.057) per 8.99 μg/m^3^ increase].

### Sensitivity analyses

Figures S4; http://links.lww.com/EE/A147 and S5; http://links.lww.com/EE/A147 show the results of sensitivity analyses with different lag structures of temperature adjustment using PM_2.5_ as an example (all other pollutants had a very similar pattern; data not shown). SA1 to SA4 generated similar RRs and 95% CIs without meaningful differences. We also observed similar single-day lag patterns to those shown in Figures 1 and 2, where lags 2 and 3 tended to have the highest RR estimates for all renal diseases and ARF, and lag 7 tended to have higher estimates for ARF. SA5 showed that the RR estimates were similar for distributed lags 0–3 and 0–7. The RRs of lag 0–7 tended to be slightly higher than those of lag 0–3, which met the expectation that exposure to air pollution for a longer period could be associated with a higher risk of kidney disease outcomes. Figure S6; http://links.lww.com/EE/A147 shows the RRs of the single-day and distributed lags of PM_2.5_ in the two-pollutant models with criteria gases. The effects of PM_2.5_ in the two-pollutant models were consistent with those in the single-pollutant models. In general, the sensitivity analysis showed similar RR estimates and lag patterns as the main analysis, indicating the robustness of both the temperature and co-pollutant adjustments.

## Discussion

We observed positive associations between ED visits for kidney disease outcomes and multiple-day exposure to PM_2.5_, major PM_2.5_ components, and gaseous co-pollutants. To the best of our knowledge, this is the first time-series study to explore associations between short-term exposure to multiple air pollutants related to PM_2.5_ and ED visits for kidney diseases in the United States.

Our study shows that compared to single-day exposure, multiple-day exposure had remarkably stronger associations with ED visits for kidney diseases (Figures 1 and 2). This result meets the expectation that exposure to air pollution in a longer period would be associated with a higher risk of kidney disease outcomes. In addition, single-day RR estimates showed a specific lag pattern: lags 2 and 3 tended to be the highest RR estimates for all renal diseases and ARF, and lag 7 tended to have higher estimates for ARF. This pattern may indicate that higher ED visits for all renal diseases were most likely to occur on 2–3 days after high levels of air pollution exposure, whereas ED visits for ARF had a more complex lagging effect with another frequent occurrence on 7 days after the exposure. The mechanism behind this lag pattern needs further explorations with more detailed kidney function-related health data (such as laboratory test data for kidney function) than ED visit records. We also observed stronger associations for ARF than all renal diseases, which may indicate that short-term exposure to air pollutants (PM_2.5_ and its components in particular) had larger and more direct effects on exacerbations of acute renal outcomes than more chronic outcomes. However, the underdiagnosis of chronic kidney diseases, as stated below, could be another possible reason for the observed stronger associations for more acute symptoms. We note that as an exploratory study conducted in a single city, we chose to examine the linear effects of short-term air pollution exposure. Further analysis for the non-linearity is important, and a multi-city dataset with larger variations in pollution concentrations and increased statistical power may be needed to support the analysis.

The analysis based on primary ED diagnosis generally showed lower RRs and larger 95% CIs than that based on any ED diagnosis (Figure 1 vs. Figure S2; http://links.lww.com/EE/A147; Figure 2 vs. Figure S3; http://links.lww.com/EE/A147), although most RRs were still greater than 1. Several reasons could explain these weaker associations. First, the primary diagnosis is less frequent and has lower statistical power. Second, as kidney problems may not generate severe kidney-related symptoms even when kidney function declines drastically (i.e., asymptomatic or paucisymptomatic),^45,46^ and other systems’ symptoms related to kidney problems might appear to be more severe, the kidney problems themselves are likely to be treated and coded as nonprimary diagnoses. In particular, kidney diseases can either be involved in or drive respiratory diseases,^47^ circulatory diseases,^48^ and diabetes.^49^ For this analysis, the distributions of the primary diagnoses of ED visits with non-primary diagnoses of kidney diseases are shown in Figure S7; http://links.lww.com/EE/A147. These distributions reflect how nonprimary diagnoses of kidney diseases relate to primary diagnoses involving in other body systems. Diseases of the circulatory system (ICD-9: 390–459) were the most frequent primary diagnosis for visits with non-primary diagnoses of kidney diseases. Diseases of the respiratory (ICD-9: 460–519) and digestive systems (ICD-9: 520–579) were also among the top-frequent primary diagnoses. Symptoms, signs, and ill-defined conditions (ICD-9: 780–799) were related to all renal diseases but not ARF. Infectious or parasitic disease (ICD-9: 001–139) was specifically associated with ARF. Underdiagnosis of kidney diseases may result in stronger observed associations of air pollution on ED visits in which kidney disease outcomes are coded as non-primary diagnoses. Finally, as kidney diseases are largely painless and without other symptoms, biases in coding the primary kidney disease diagnosis could be another possible reason. Additional studies are needed to quantify the extent of underdiagnosis with the use of ED visit records.

Previous epidemiological studies have demonstrated positive associations between long-term exposure to PM_2.5_ and increased risks of kidney disease outcomes.^10–14^ PM_2.5_-associated kidney disease outcomes mainly included incident CKD, eGFR decline, and new diagnosis of ESRD in these studies. Long-term exposure to PM_2.5_ was also found to be significantly associated with an increased risk of membranous nephropathy^12^ as well as kidney disease mortality.^11^ Several recent epidemiological studies assessed the effects of short-term exposure to PM_2.5_ on kidney diseases/functions in China^20–22^ and the United States.^23^ These studies had different scopes from our analysis, which focused on specific age groups, such as the elderly^20,23^ and children,^22^ or hospital admission.^21,23^ Statistically significant associations between short-term exposure to PM_2.5_ and decline of kidney function or increased risks of kidney disease hospital admissions were observed in these studies. For example, a panel study with 76 participants 60–69 years of age in Jinan, China observed that significant declines in eGFR from −1.69% (95% CI: −3.34%, −0.01%) to −3.27% (95% CI: −5.04%, −1.47%) were associated with IQR increases in personal PM_2.5_ exposures at different lag periods (from 7–12 to 49–72 hours).^20^ Another panel study with 105 children 4–13 years of age in Wuhan, China, showed that personal exposure to PM_2.5_ was dose-responsive related to decline in eGFR within lag 2 days with the strongest at lag 0 day [−1.69% (95% CI: −2.27%, −1.10%) in eGFR per 10 μg/m^3^ increase in PM_2.5_].^22^ Gu et al^21^ and Wei et al^23^ reported that short-term (daily) PM_2.5_ exposure was associated with increased risks of hospital admission for kidney diseases based on electronic inpatient records in China (all-age) and the United States (age 65 years or older), respectively.

A growing number of toxicological results have revealed possible mechanisms of PM_2.5_ affecting kidney functions.^5–8,50,51^ These toxicological studies suggest that inhaled ultrafine particles can affect distant organs, including kidneys, in either direct or indirect ways. First, the inhaled particles could provoke pulmonary inflammation which may then indirectly lead to inflammation in kidneys.^51^ Second, as the kidney is a vascularized organ, biological hypotheses related to the associations between PM_2.5_ exposure and cardiovascular disease outcomes may also be pertinent to kidney disease outcomes.^10,14,52^ One possibility is that fine particle-induced disturbances in the pulmonary nervous system could affect the function of the heart, causing congestion of blood in kidneys.^52^ Another possibility is that fine particles entering the bloodstream would directly interact with the kidney vasculature to promote the decline of kidney function. Last, a mechanism is supported by Miller et al^9^ who found that inert gold nanoparticles could be detected in the urine within minutes after exposure, which indicates the possibility of fine particles directly entering kidneys and influencing kidney function. In general, both epidemiological and toxicological evidence show possible rationales for the associations observed in this study.

There are several limitations to this study. First, although the use of the CMAQ-fused air pollution exposure estimates is an improvement over the use of measurements from routine centrally located air monitoring stations, prediction errors in the fusion model and Berkson-type errors due to spatial aggregation of pollution levels could potentially impact the observed exposure-outcome relationships. These errors are likely to differ among different air pollutants according to their spatiotemporal variability. Simulation studies have shown that this source of exposure measurement error in time-series analyses often leads to bias toward the null.^35,53^ Additionally, as we examined associations between kidney disease outcomes and short-term exposure to several air pollutants, some statistically significant associations may have been observed by chance. Moreover, the study only utilized ED visit billing records, which might be subject to ascertainment bias. However, this ascertainment bias is not likely related to air pollution levels, and is not expected to bias the magnitude of air pollution effect estimates. More precise metrics of the presence and severity of kidney diseases other than ICD-9 code diagnoses (e.g., eGFR and urinary indicators) may be useful for more accurate case ascertainment. Furthermore, the exclusion of ESRD patients on dialysis may be inadequate because of the incorrect diagnosis codes. For example, some diabetic renal disease patients might also be on dialysis, but it was not possible to identify these patients in our ED visit records. Finally, since this study was conducted in Atlanta, the observed PM_2.5_-kidney disease associations need further validations in different geographical areas and for different populations.

## Conclusions

This study assessed the relative risk of ED visits for kidney diseases associated with short-term exposure to PM_2.5_, major PM_2.5_ components, and gaseous co-pollutants in Atlanta over a 7-year period. We observed positive associations between short-term exposure to fine particulate air pollution and kidney disease outcomes, ARF in particular. This study adds to the growing epidemiological evidence that fine particles may impact distant organs such as kidneys over the short term. The observed PM_2.5_-kidney disease relationships warrant further validations in other geographical regions.

## Conflicts of interest statement

The authors declare that they have no conflicts of interest with regard to the content of this report.

## ACKNOWLEDGMENTS

We are grateful for our fruitful cooperation with the Georgia Hospital Association and the support of all participating hospitals.

## Supplementary Material


