# Supplementary material for: Short-term exposure to fine particulate air pollution and emergency department visits for kidney diseases in the Atlanta metropolitan area
Source: Environ Epidemiol. 2021 Aug 6;5(4):e164. doi: 10.1097/EE9.0000000000000164 (PMC8367053; doi:10.1097/EE9.0000000000000164)
Supplement: Supplementary file 1 [file ee9-5-e164-s001.pdf]

Supplemental Materials

Short-term exposure to fine particulate air pollution and emergency department visits for kidney diseases in the Atlanta metropolitan area

Bi, Jianzhao[1,*]; Barry, Vaughn[2]; Weil, Ethel J[3]; Chang, Howard H[4]; Ebelt, Stefanie[2]

[1]Department of Environmental & Occupational Health Sciences, School of Public Health, University of Washington, Seattle, WA

[2]Gangarosa Department of Environmental Health, Rollins School of Public Health, Emory University, Atlanta, GA

[3]Department of Medicine, School of Medicine, Emory University, Atlanta, GA

[4]Department of Biostatistics and Bioinformatics, Rollins School of Public Health, Emory University, Atlanta, GA

[*]Corresponding Author. Address: Department of Environmental & Occupational Health Sciences, School of Public Health, University of Washington, 4225 Roosevelt Way NE, Seattle, WA 98105. E-mail: jbi6@uw.edu (J. Bi).

Table S1: Structures of five sensitivity analyses (SAs) for daily maximum and minimum air temperature and mean dew-point temperature with cubic (CU) terms (the main models only adjusted for cubic terms for daily maximum air temperature and mean dew-point temperature). The SAs included 1) SA1: lag 0 of maximum air temperature, moving average from lag 1 to the corresponding lag of minimum air temperature, and moving average from lag 0 to the corresponding lag of dew-point temperature, 2) SA2: lag 0 of maximum air temperature, moving average from lag 1 to the corresponding lag of maximum air temperature, and moving average from lag 0 to the corresponding lag of dew-point temperature, 3) SA3: moving averages from lag 0 to the corresponding lag of maximum air temperature and dew-point temperature, and 4) SA4: single-day lags of maximum air temperature and dew-point temperature, and 5) SA5: comparing the distributed lag 0-7 (the cumulative effect of air pollution exposure over eight days) with the distributed lag 0-3 (the cumulative effect of air pollution exposure over four days). SA1 to SA4 were employed to validate the single-day lag models; SA5 was employed to validate the distributed lag models.

Sensitivity Analysis	Air Pollution Term	Maximum Temp Terms (CU)	Minimum Temp Terms (CU)	Dew-point Temp Terms (CU)
SA 1 (Single-Day Lag)	Lag M*	Lag 0	Lag 1-M'**	Lag 0-M
SA 2 (Single-Day Lag)	Lag M	Lag 0 / Lag 1-M'	--	Lag 0-M
SA 3 (Single-Day Lag)	Lag M	Lag 0-M	--	Lag 0-M
SA 4 (Single-Day Lag)	Lag M	Lag M	--	Lag M
SA 5 (Distributed Lag)	Lags 0-3 and 0-7	Lags 0-3 and 0-7	--	Lags 0-3 and 0-7

* $M \in 0, 1, 2, 3, 4, 5, 6, 7$
** $M' \in 1, 2, 3, 4, 5, 6, 7$

Table S2: Single-day lag RRs and 95% CIs of the associations between short-term exposure to air pollution and ED visits for kidney diseases.

Pollutant	IQR	Lag	All Renal Diseases, Primary Diagnosis RR (95% CI)	All Renal Diseases, Any Diagnosis RR (95% CI)	ARF, Primary Diagnosis RR (95% CI)	ARF, Any Diagnosis RR (95% CI)
O_3	27.04 ppb	0	0.988 (0.966, 1.010)	0.989 (0.974, 1.004)	1.018 (0.954, 1.085)	0.987 (0.958, 1.017)
		1	1.012 (0.990, 1.035)	1.015 (1.000, 1.031)*	1.014 (0.951, 1.082)	1.029 (0.998, 1.060)*
		2	1.007 (0.985, 1.030)	1.008 (0.993, 1.024)	0.987 (0.925, 1.053)	1.027 (0.997, 1.059)*
		3	1.006 (0.983, 1.029)	1.012 (0.996, 1.027)	1.007 (0.944, 1.075)	1.039 (1.008, 1.071)**
		4	0.994 (0.971, 1.016)	0.991 (0.976, 1.007)	1.023 (0.958, 1.093)	1.004 (0.974, 1.035)
		5	0.985 (0.963, 1.008)	0.985 (0.970, 1.000)*	0.976 (0.914, 1.043)	0.985 (0.956, 1.016)
		6	0.987 (0.964, 1.009)	0.995 (0.979, 1.010)	0.988 (0.926, 1.055)	1.010 (0.980, 1.041)
		7	1.003 (0.980, 1.026)	1.006 (0.991, 1.022)	1.059 (0.992, 1.130)*	1.024 (0.994, 1.056)
CO	0.33 ppm	0	0.993 (0.983, 1.002)	0.992 (0.986, 0.998)**	0.993 (0.966, 1.020)	0.984 (0.972, 0.997)**
		1	1.006 (0.997, 1.016)	1.002 (0.996, 1.009)	1.003 (0.976, 1.031)	0.999 (0.987, 1.012)
		2	1.011 (1.002, 1.021)**	1.006 (1.000, 1.013)*	1.017 (0.990, 1.046)	1.007 (0.995, 1.020)
		3	1.005 (0.996, 1.015)	1.003 (0.996, 1.009)	1.012 (0.985, 1.040)	1.012 (0.999, 1.024)*
		4	0.997 (0.987, 1.006)	1.003 (0.996, 1.009)	0.980 (0.954, 1.007)	1.006 (0.994, 1.018)
		5	0.997 (0.987, 1.006)	1.000 (0.994, 1.007)	0.986 (0.959, 1.014)	0.997 (0.985, 1.009)
		6	1.005 (0.995, 1.014)	1.004 (0.998, 1.011)	1.001 (0.974, 1.029)	0.999 (0.986, 1.011)
		7	0.999 (0.990, 1.009)	1.003 (0.997, 1.009)	1.023 (0.995, 1.051)	1.010 (0.998, 1.022)
SO_2	8.76 ppb	0	1.008 (0.999, 1.017)*	1.004 (0.998, 1.010)	1.009 (0.985, 1.034)	0.998 (0.986, 1.009)
		1	0.997 (0.988, 1.006)	0.998 (0.992, 1.004)	0.993 (0.969, 1.018)	0.999 (0.988, 1.011)
		2	0.996 (0.987, 1.005)	0.995 (0.989, 1.001)	0.997 (0.972, 1.022)	0.991 (0.980, 1.003)
		3	1.002 (0.993, 1.011)	0.996 (0.990, 1.002)	0.997 (0.972, 1.022)	0.997 (0.986, 1.009)
		4	1.002 (0.993, 1.011)	0.997 (0.991, 1.003)	0.988 (0.963, 1.013)	1.000 (0.988, 1.011)
		5	1.009 (1.000, 1.018)*	1.003 (0.997, 1.009)	1.007 (0.982, 1.032)	1.002 (0.991, 1.014)
		6	0.996 (0.987, 1.005)	0.999 (0.993, 1.005)	0.994 (0.970, 1.020)	1.002 (0.990, 1.014)
		7	1.005 (0.996, 1.014)	1.003 (0.997, 1.009)	1.015 (0.990, 1.041)	1.008 (0.996, 1.019)
NO_2	9.52 ppb	0	0.994 (0.983, 1.007)	0.993 (0.985, 1.001)	0.991 (0.959, 1.025)	0.981 (0.966, 0.996)**
		1	1.008 (0.996, 1.020)	1.003 (0.995, 1.011)	1.014 (0.980, 1.048)	1.002 (0.987, 1.017)
		2	1.017 (1.005, 1.029)**	1.007 (0.999, 1.015)	1.022 (0.988, 1.057)	1.012 (0.997, 1.027)
		3	1.014 (1.002, 1.027)**	1.004 (0.996, 1.013)	0.997 (0.964, 1.031)	1.007 (0.992, 1.023)
		4	0.995 (0.983, 1.007)	0.999 (0.991, 1.007)	0.971 (0.940, 1.004)*	1.000 (0.985, 1.015)
		5	0.998 (0.986, 1.010)	0.999 (0.991, 1.007)	0.969 (0.937, 1.002)*	0.989 (0.975, 1.005)
		6	1.007 (0.994, 1.019)	1.004 (0.996, 1.012)	1.012 (0.979, 1.046)	1.000 (0.985, 1.015)
		7	0.997 (0.985, 1.009)	1.002 (0.994, 1.010)	1.024 (0.991, 1.059)	1.008 (0.993, 1.023)
NO_x	33.51 ppb	0	0.994 (0.985, 1.004)	0.994 (0.987, 1.000)*	0.984 (0.958, 1.010)	0.987 (0.976, 0.999)**
		1	1.005 (0.996, 1.014)	1.003 (0.997, 1.009)	1.004 (0.978, 1.031)	1.001 (0.989, 1.013)
		2	1.010 (1.001, 1.019)**	1.005 (0.999, 1.011)	1.019 (0.993, 1.047)	1.009 (0.997, 1.021)

		3	1.009 (1.000, 1.019)*	1.002 (0.996, 1.009)	1.014 (0.988, 1.041)	1.008 (0.996, 1.020)
		4	0.999 (0.990, 1.008)	1.001 (0.995, 1.007)	0.978 (0.953, 1.005)	1.005 (0.993, 1.017)
		5	0.998 (0.989, 1.008)	0.999 (0.993, 1.005)	0.987 (0.961, 1.013)	0.998 (0.986, 1.009)
		6	1.004 (0.995, 1.014)	1.004 (0.997, 1.010)	1.005 (0.979, 1.032)	1.000 (0.988, 1.012)
		7	0.999 (0.989, 1.008)	1.001 (0.995, 1.007)	1.020 (0.994, 1.047)	1.008 (0.996, 1.019)
$PM_{2.5}$	8.99 μg/m^3	0	0.990 (0.981, 1.000)*	0.991 (0.985, 0.998)**	0.996 (0.969, 1.024)	0.997 (0.984, 1.010)
		1	1.004 (0.995, 1.014)	1.002 (0.995, 1.009)	1.007 (0.980, 1.035)	1.008 (0.995, 1.021)
		2	1.008 (0.998, 1.018)	1.005 (0.999, 1.012)	0.993 (0.966, 1.021)	1.015 (1.002, 1.028)**
		3	1.008 (0.998, 1.018)	1.006 (0.999, 1.013)	1.001 (0.974, 1.029)	1.011 (0.998, 1.024)
		4	0.995 (0.985, 1.004)	0.995 (0.989, 1.002)	0.979 (0.953, 1.007)	0.996 (0.983, 1.009)
		5	1.000 (0.990, 1.010)	0.999 (0.992, 1.006)	0.997 (0.970, 1.025)	1.001 (0.988, 1.014)
		6	1.006 (0.997, 1.016)	1.004 (0.998, 1.011)	1.012 (0.984, 1.040)	1.003 (0.991, 1.016)
		7	1.002 (0.992, 1.012)	1.001 (0.994, 1.008)	1.009 (0.982, 1.037)	0.999 (0.987, 1.012)
EC	0.68 μg/m^3	0	0.992 (0.983, 1.001)*	0.994 (0.988, 1.000)*	0.986 (0.962, 1.011)	0.992 (0.981, 1.003)
		1	1.005 (0.996, 1.014)	1.002 (0.996, 1.008)	1.003 (0.978, 1.029)	1.002 (0.991, 1.014)
		2	1.013 (1.004, 1.022)**	1.007 (1.001, 1.013)**	1.019 (0.993, 1.045)	1.013 (1.001, 1.024)**
		3	1.006 (0.997, 1.015)	1.003 (0.997, 1.009)	1.017 (0.991, 1.042)	1.014 (1.003, 1.026)**
		4	0.995 (0.987, 1.004)	0.999 (0.993, 1.005)	0.976 (0.952, 1.001)*	1.001 (0.990, 1.013)
		5	1.003 (0.994, 1.012)	0.999 (0.993, 1.005)	0.993 (0.969, 1.019)	0.995 (0.984, 1.006)
		6	1.004 (0.995, 1.014)	1.004 (0.998, 1.010)	1.011 (0.986, 1.037)	1.000 (0.989, 1.012)
		7	1.005 (0.996, 1.014)	1.004 (0.998, 1.010)	1.018 (0.993, 1.043)	1.008 (0.997, 1.019)
OC	1.75 μg/m^3	0	0.993 (0.985, 1.001)	0.994 (0.989, 1.000)*	0.992 (0.971, 1.015)	0.995 (0.985, 1.006)
		1	1.003 (0.995, 1.011)	1.000 (0.995, 1.006)	0.993 (0.971, 1.016)	1.004 (0.994, 1.014)
		2	1.009 (1.001, 1.018)**	1.006 (1.001, 1.011)**	1.012 (0.990, 1.034)	1.011 (1.001, 1.022)**
		3	1.006 (0.998, 1.014)	1.005 (1.000, 1.011)*	1.011 (0.989, 1.033)	1.013 (1.003, 1.023)**
		4	0.993 (0.985, 1.001)	0.999 (0.993, 1.004)	0.972 (0.950, 0.994)**	1.000 (0.990, 1.010)
		5	0.999 (0.991, 1.008)	0.999 (0.994, 1.005)	0.990 (0.968, 1.013)	0.999 (0.988, 1.009)
		6	1.004 (0.995, 1.012)	1.004 (0.999, 1.010)	1.000 (0.978, 1.023)	1.003 (0.992, 1.013)
		7	1.004 (0.996, 1.012)	1.005 (0.999, 1.010)	1.013 (0.991, 1.035)	1.004 (0.994, 1.015)
Sulfate	3.52 μg/m^3	0	0.992 (0.982, 1.002)	0.994 (0.987, 1.000)*	1.008 (0.980, 1.037)	1.003 (0.990, 1.017)
		1	1.000 (0.990, 1.009)	0.998 (0.992, 1.005)	1.008 (0.980, 1.036)	1.005 (0.992, 1.019)
		2	0.997 (0.987, 1.006)	0.997 (0.991, 1.004)	0.991 (0.963, 1.020)	1.009 (0.996, 1.023)
		3	1.005 (0.996, 1.015)	1.002 (0.996, 1.009)	1.007 (0.979, 1.036)	1.010 (0.997, 1.024)
		4	0.997 (0.988, 1.007)	0.995 (0.988, 1.001)	0.989 (0.961, 1.018)	0.994 (0.981, 1.007)
		5	0.999 (0.989, 1.009)	1.000 (0.993, 1.006)	1.006 (0.978, 1.036)	1.006 (0.992, 1.019)
		6	1.003 (0.993, 1.013)	1.001 (0.994, 1.007)	1.006 (0.978, 1.035)	1.001 (0.988, 1.015)
		7	1.002 (0.992, 1.012)	0.997 (0.991, 1.004)	1.019 (0.990, 1.048)	0.997 (0.983, 1.010)
Nitrate	0.60 μg/m^3	0	0.999 (0.989, 1.009)	0.996 (0.990, 1.003)	1.011 (0.983, 1.039)	0.995 (0.982, 1.007)
		1	1.003 (0.993, 1.013)	1.002 (0.996, 1.009)	0.996 (0.968, 1.024)	1.002 (0.990, 1.015)

		2	1.005 (0.996, 1.015)	1.001 (0.995, 1.008)	0.987 (0.960, 1.015)	0.998 (0.985, 1.011)
		3	1.005 (0.995, 1.015)	1.003 (0.996, 1.010)	0.998 (0.971, 1.027)	1.003 (0.990, 1.016)
		4	1.006 (0.996, 1.016)	1.001 (0.995, 1.008)	1.008 (0.980, 1.036)	1.003 (0.990, 1.016)
		5	1.001 (0.992, 1.011)	1.001 (0.995, 1.008)	0.998 (0.970, 1.026)	1.004 (0.992, 1.017)
		6	1.005 (0.995, 1.015)	1.001 (0.995, 1.008)	1.020 (0.992, 1.048)	1.004 (0.992, 1.017)
		7	0.996 (0.986, 1.006)	0.998 (0.992, 1.005)	1.004 (0.976, 1.032)	1.004 (0.992, 1.017)

* 0.05 <= p < 0.10; ** p < 0.05

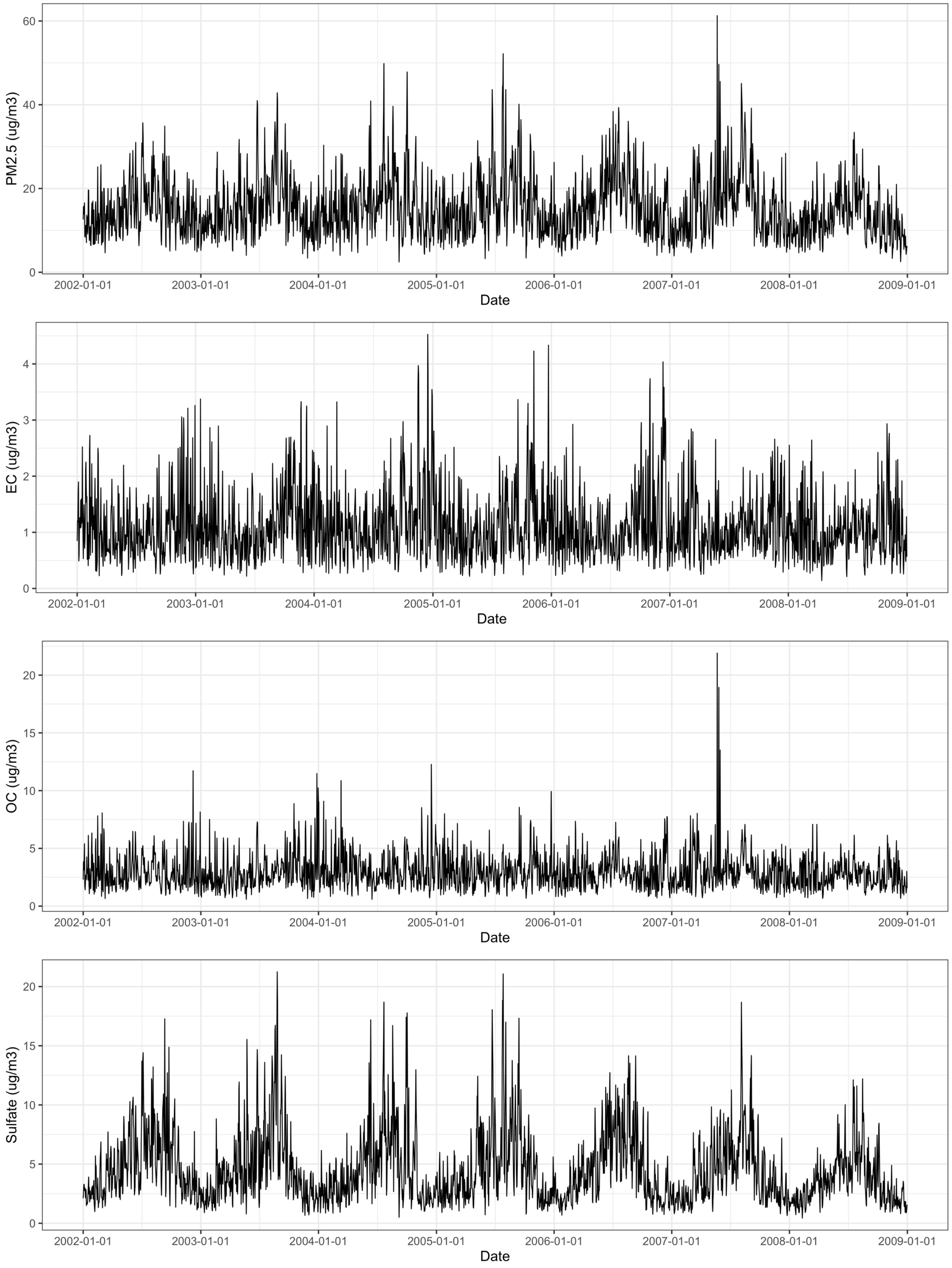
PM2.5 (ug/m3)
0
20
40
60
EC (ug/m3)
0
1
2
3
4
OC (ug/m3)
0
5
10
15
20
Sulfate (ug/m3)
0
5
10
15
20
2002-01-01
2003-01-01
2004-01-01
2005-01-01
2006-01-01
2007-01-01
2008-01-01
2009-01-01
Date

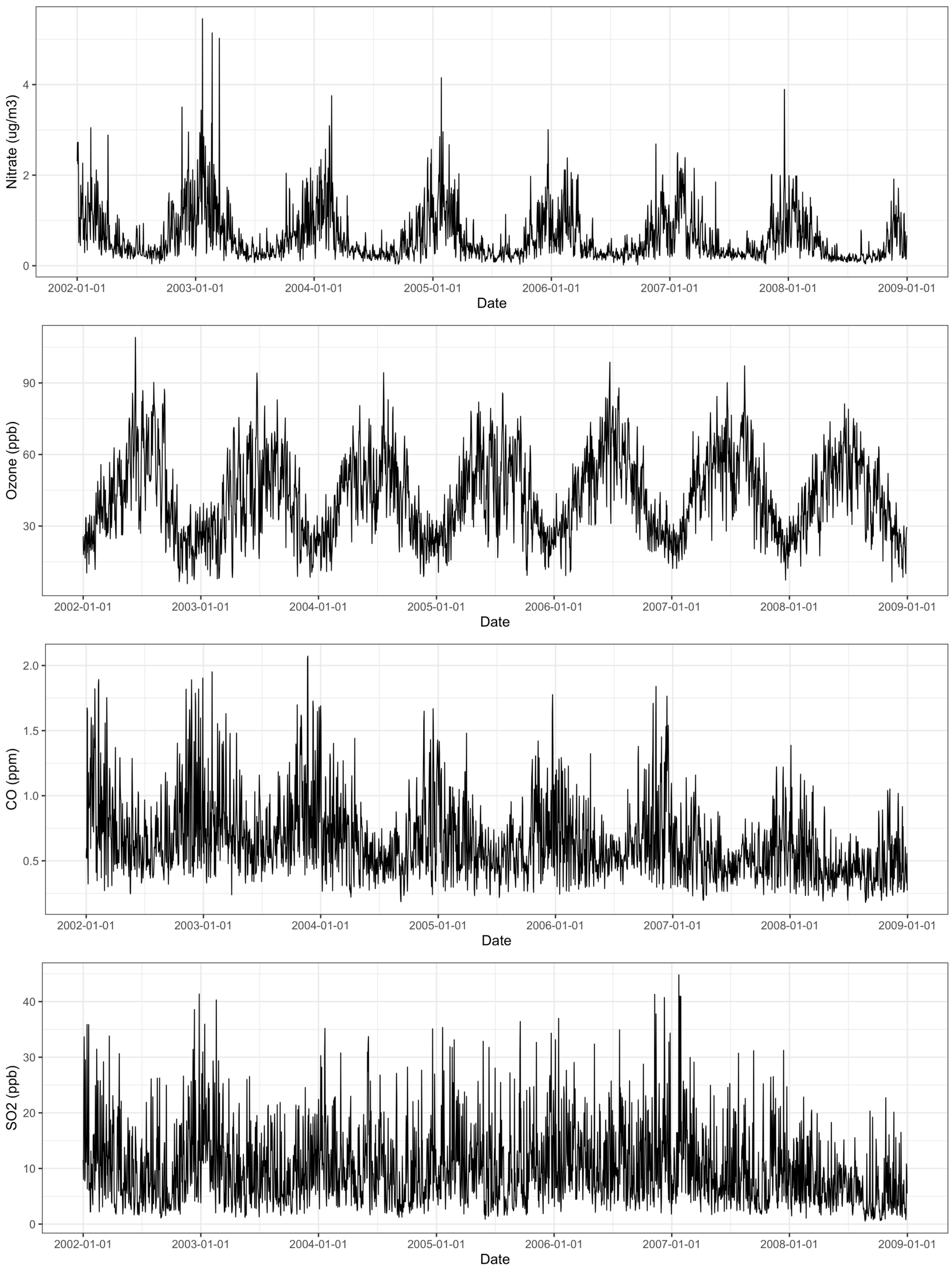
Nitrate (ug/m3)
0
2
4
Ozone (ppb)
30
60
90
CO (ppm)
0.5
1.0
1.5
2.0
SO2 (ppb)
0
10
20
30
40
2002-01-01
2003-01-01
2004-01-01
2005-01-01
2006-01-01
2007-01-01
2008-01-01
2009-01-01
Date

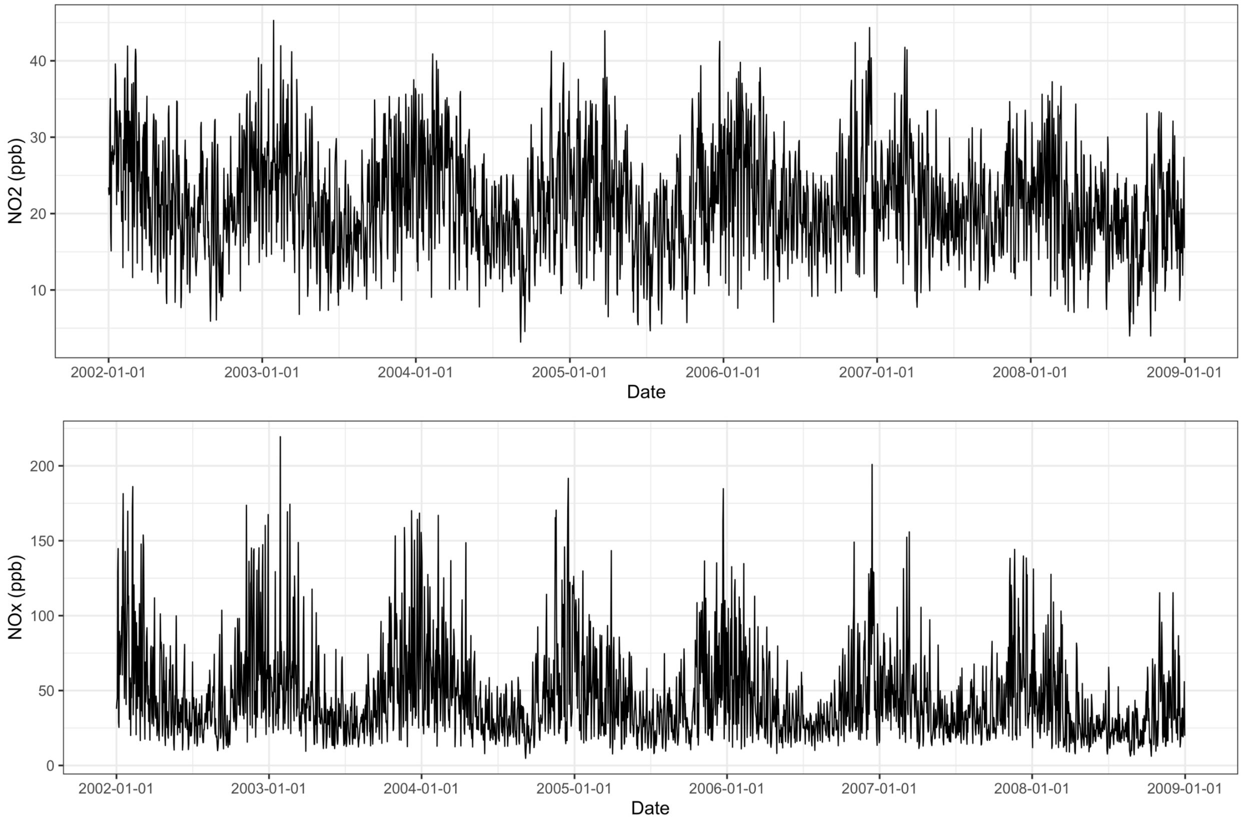

Figure S1: Time-series of air pollution concentrations in Atlanta from the CMAQ-fused model.

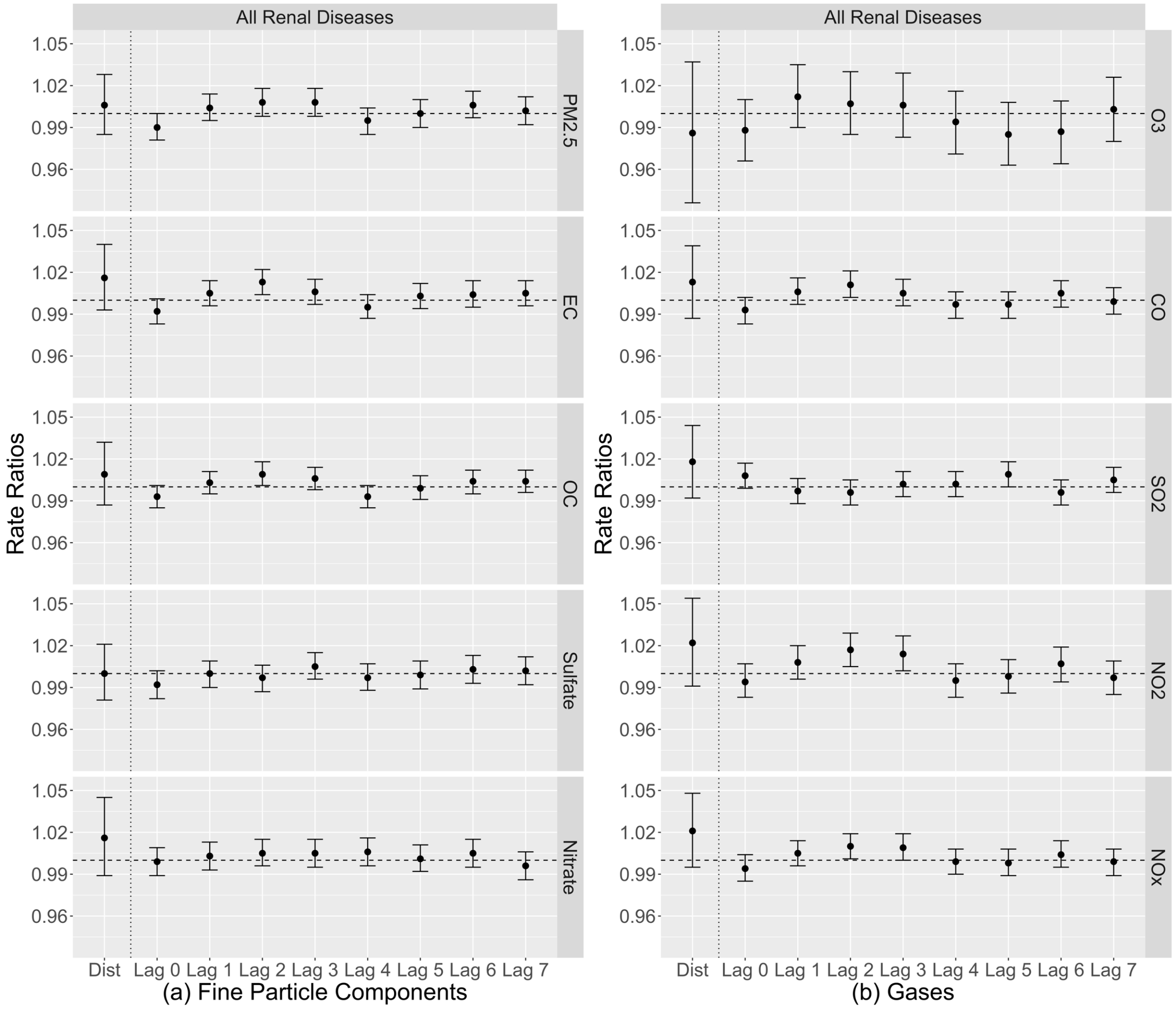

Figure S2: RR estimates of ED visits (primary diagnosis) for all renal diseases associated with short-term air pollution exposure (left: fine particle components; right: criteria gases) with different lag structures (distributed lag [dist.] and single-day lags) in Atlanta during the period of 2002-2008.

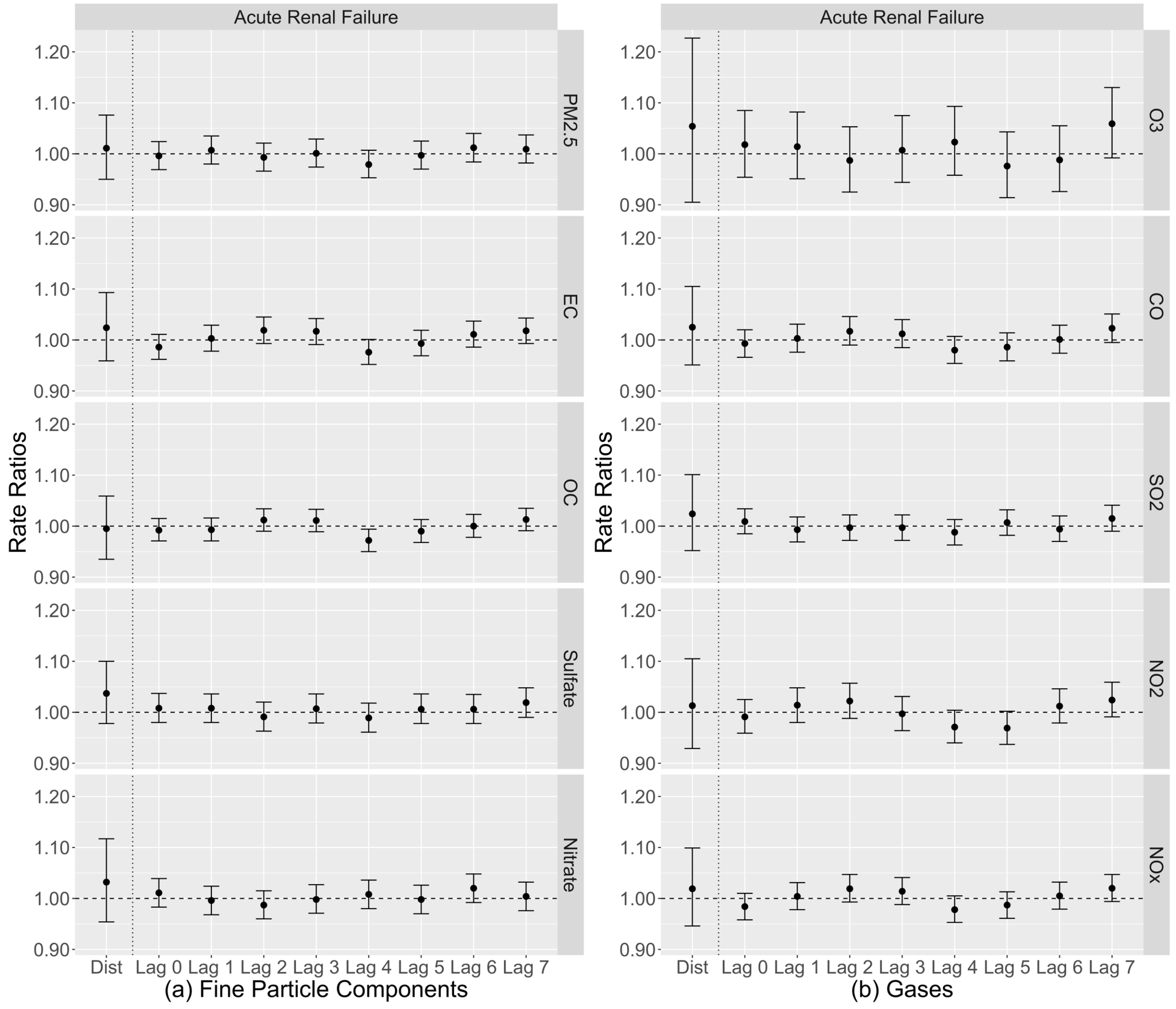

Figure S3: RR estimates of ED visits (primary diagnosis) for ARF associated with short-term air pollution exposure (left: fine particle components; right: criteria gases) with different lag structures (distributed lag [dist.] and single-day lags) in Atlanta during the period of 2002-2008.

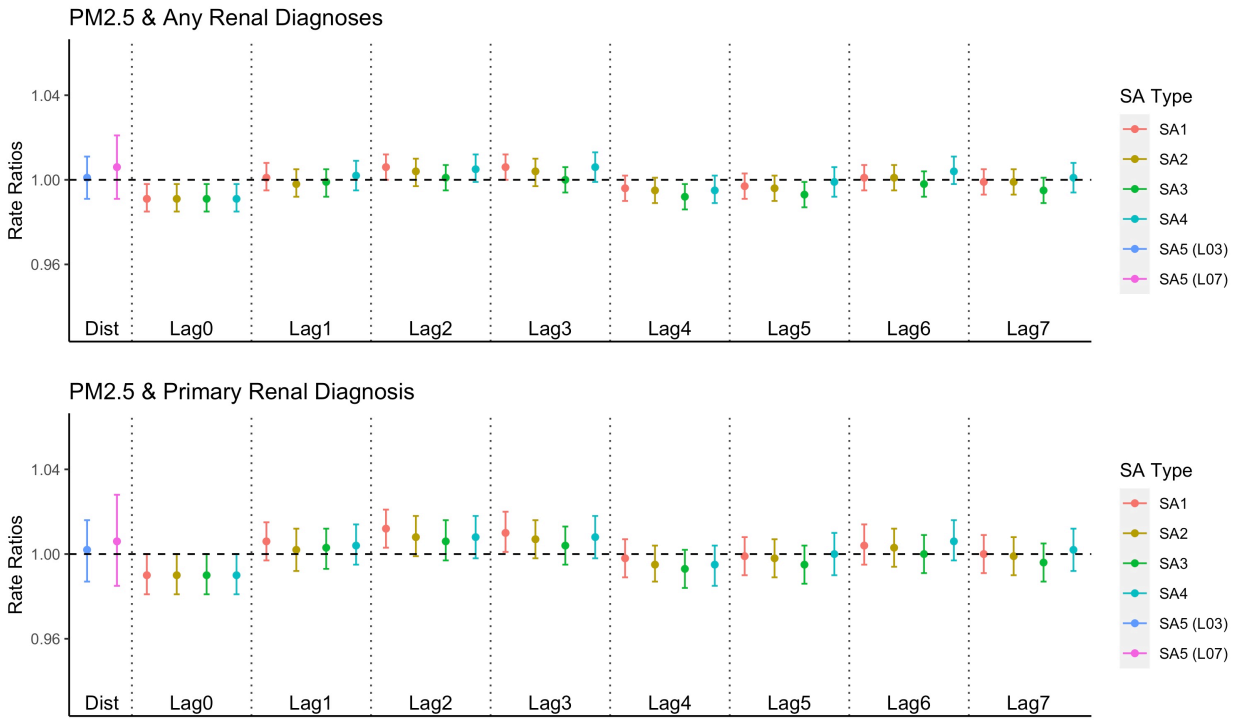

Figure S4: Sensitivity analysis of temperature adjustment for the RR estimates of primary and any ED visits for all renal diseases associated with short-term exposure to air pollution ($PM_{2.5}$ as an example).

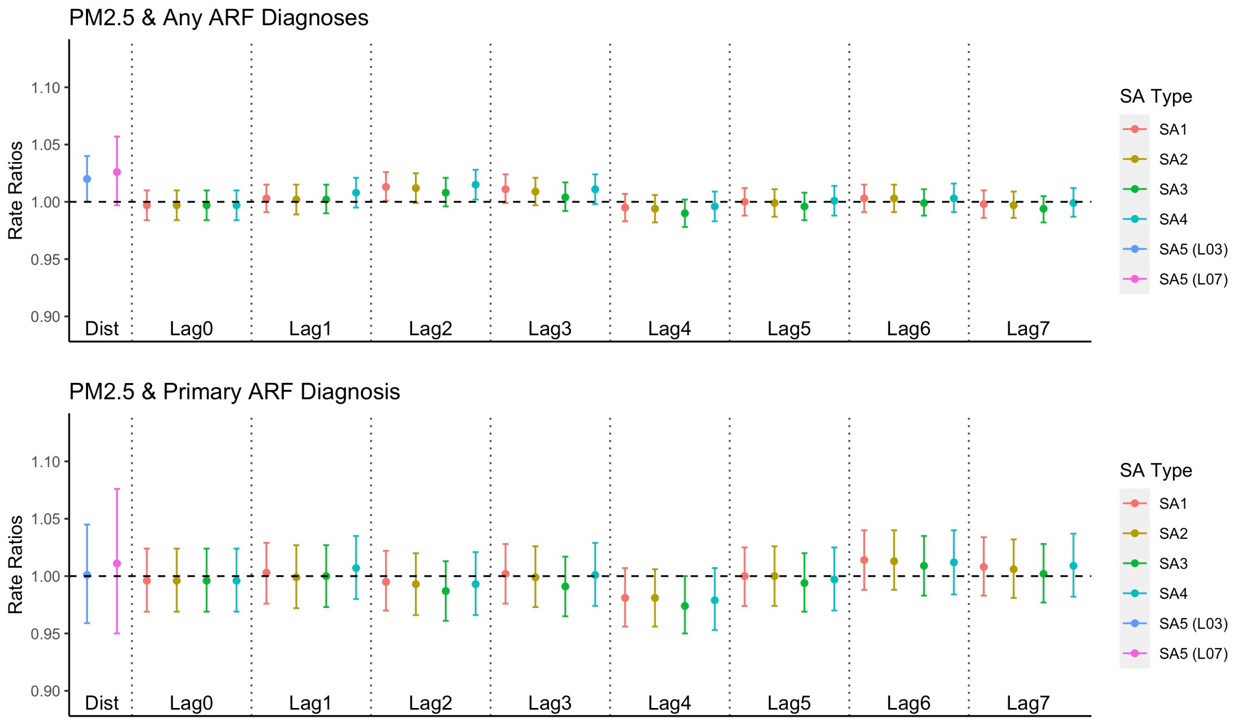

Figure S5: Sensitivity analysis of temperature adjustment for the RR estimates of primary and any ED visits for ARF associated with short-term exposure to air pollution ($PM_{2.5}$ as an example).

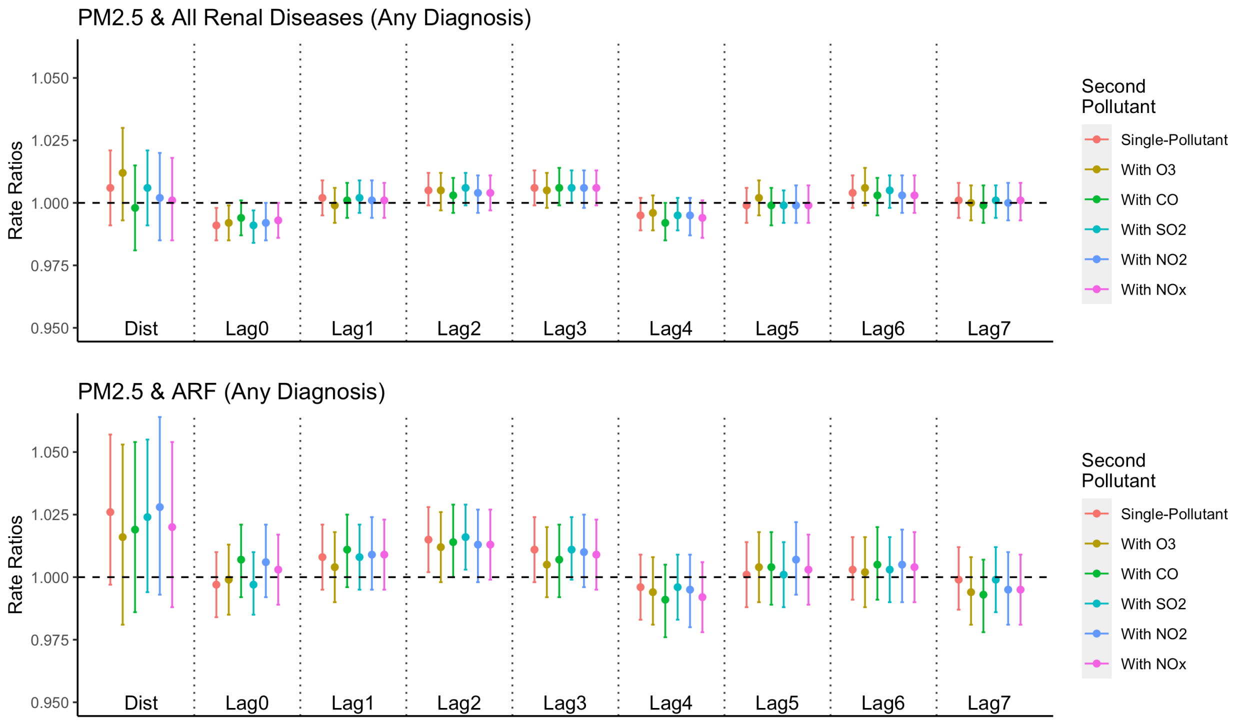

Figure S6: Sensitivity analysis of two-pollutant models with criteria gases one at a time for the RR estimates of ED visits for both all renal diseases and ARF associated with short-term exposure to $PM_{2.5}$.

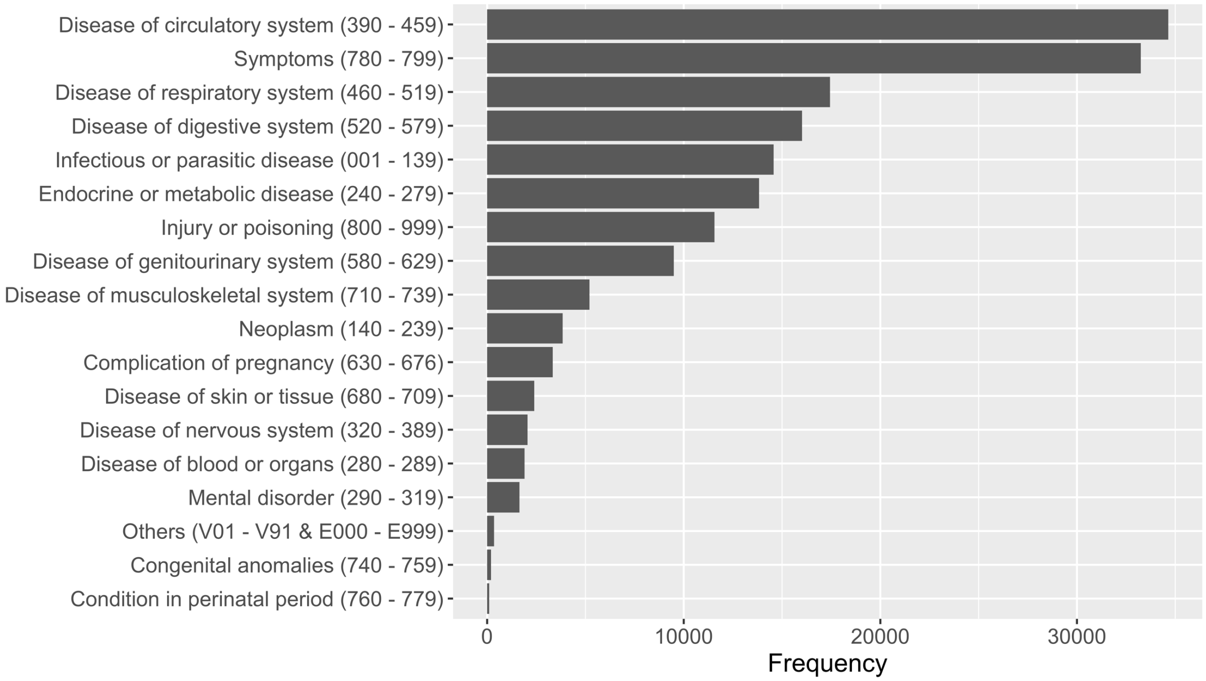

(a) All Renal Diseases

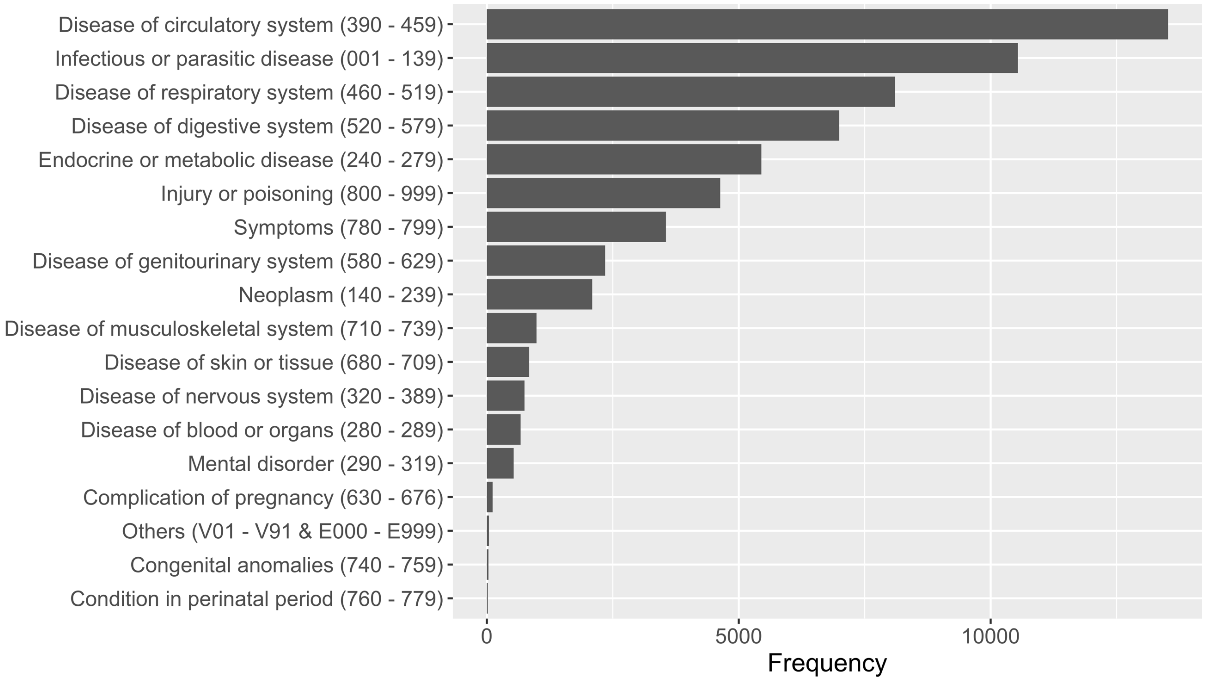

(b) ARF

Figure S7: Distributions of primary diagnoses of ED visits with non-primary diagnoses of kidney diseases: (a) all renal diseases; (b) ARF. The diagnosis classification was based on the ICD-9 codes.
